# A novel writing to dictation test to investigate lexical and sublexical processes: Italian normative data and preliminary clinical feasibility in Mild Cognitive Impairment and Alzheimer’s disease

**DOI:** 10.1007/s10072-026-09256-1

**Published:** 2026-07-17

**Authors:** Beatrice Feder, Giulia Polli, Susanna Laurenzi, Serena Righetti, Barbara Tessari, Stefano Terruzzi, Costanza Papagno

**Affiliations:** 1 Center for Cognitive Neurorehabilitation (CeRiN), Center for Mind/Brain Sciences (CIMeC), University of Trento–via Matteo Del Ben, 5/b, Rovereto, TN 38068 Italy; 2 Department of Psychology, University of Milano-Bicocca, Piazza dell’Ateneo Nuovo 1, Milan, 20126 Italy

**Keywords:** Spelling assessment, Lexical and sub-lexical processing, Mild Cognitive Impairment (MCI), Alzheimer’s Disease (AD), Neuropsychological evaluation

## Abstract

Writing abilities can be interpreted within dual-route models, which distinguish lexical and sublexical processes. The present study aimed to validate a novel writing to dictation test to assess both lexical and sub-lexical spelling abilities in an Italian population. A sample of 392 healthy participants provided robust normative data, revealing significant effects of age, education, and sex on spelling performance. Test–retest analyses indicated satisfactory reliability and minimal learning effects. The clinical feasibility of the test was explored in patients with amnestic Mild Cognitive Impairment (MCI) and Alzheimer’s disease (AD). MCI patients mainly showed selective difficulties in spelling words with unpredictable orthography, suggesting early lexical impairment, whereas AD patients exhibited more severe and heterogeneous deficits involving both lexical and sublexical processes. Qualitative error analysis revealed multiple dysgraphic patterns, particularly in the AD group. Spelling performance did not significantly correlate with other cognitive domains, which may suggest a relative specificity of writing impairment. While the test provides a useful tool for assessing spelling abilities and characterizing writing to dictation disorders in neurodegenerative conditions, limitations related to sample stratification and socio-demographic representation were noted. The study underscores the importance of including standardized writing to dictation assessments in neuropsychological evaluations.

**Supplementary Information:**

The online version contains supplementary material available at 10.1007/s10072-026-09256-1.

## Introduction

Spelling performance of neurologically unimpaired adults can be described by dual route models [e.g., 1, 2]. These models assume the existence of two independent pathways: a lexical and a sublexical-level route. The former is based on the access to the stored lexical-semantic representations, the latter on phoneme-to-grapheme conversion rules. The lexical route allows one to spell regular and irregular words whose orthography is known. Sublexical-level procedures allow instead to spell new words and regular words. The obtained orthographic string is then stored in the graphemic buffer, a short-term memory store, before being addressed to peripheral output procedures and subsequent handwriting or typing motor control [3].

Single word spelling tasks allow to establish the specific writing impairment in relation to the dual route models. A damage to the central levels of writing involves the phonologic (and)/or the semantic spelling systems (*‘central dysgraphia*’). Observation of patients suffering from an acquired brain lesion showed that some of them cannot access lexical knowledge but still correctly apply the phonological-to-orthographic conversion rules, thus making phonologically plausible errors when writing irregular words (*‘surface dysgraphia*’) [e.g., 4, 5]. Other patients can write words they have learned to spell in the past, whether regular or irregular, but are unable to write a legal non-word (*‘phonological dysgraphia*’) [e.g., 1, 2, 6]. In contrast, an impairment of the graphemic buffer causes a spelling disorder affecting both retrieved and assembled orthographic strings, with word length effect being the most characteristic feature [7]. Finally, dysgraphia may occur from damage beyond the graphemic buffer (*‘peripheral dysgraphia*’), and involve the retrieval of letter shapes, and the corresponding hand-writing motor programs, and letter names [8].

For the Italian language, one single word spelling task to investigate writing abilities in relation to the dual route models has been developed by Luzzatti et al. [9]. This battery includes five sections for a total of 183 stimuli: (i) regular words with complete one-sound-to-one-letter correspondence, (ii) words with a non-one-to-one phoneme-grapheme correspondence, whose spelling follows a complex regular pattern, (iii) words with unpredictable transcription along the phonological-to-orthographic conversion route, (iv) words that are loanwords from other languages but widely integrated into the Italian lexicon and (v) non-words with one-sound-to-one letter correspondence. Normative values were obtained from a sample of 110 Italian healthy participants with at least 8 years of education. Furthermore, Luzzatti and co-workers [10], using three out of the five sections of the battery (namely, regular words, unpredictable words, and non-words), compared the performance of 23 patients suffering from Alzheimer’s Disease (AD) to that of a group of 20 Italian healthy controls. Indeed, writing disorders have been investigated not only among patients suffering from an acquired focal brain lesion, but also among different neurodegenerative diseases. Several studies suggest that the central aspects may be involved in the very first stages of the disease while peripheral deficits would show up with the progression of dementia [11, for review]: one hypothesis is that surface dysgraphia may be the most typical writing disorder at AD onset while, in the later stages of the disease, patients would tend to produce an increasing number of non-phonologically plausible errors (substitutions, omissions and perseverations), and peripheral graphomotor errors [e.g., 12–14]. However, this hypothesis has been questioned by other studies reporting multiple patterns of writing disorders in AD [15, for review]. According to the latter hypothesis, Luzzatti et al., [10] found that AD patients spanned the whole spectrum of dysgraphia taxonomy. Moreover, nine out of the 23 patients were re-tested by the authors after an interval of 6–12 months: data confirmed the involvement of the orthographic pathway at the sublexical level not only at later stages of AD, but also at the early and intermediate ones, with high variability across individuals.

Other single word spelling tasks are included in batteries that evaluate the main features of language in patients suffering from an acquired brain lesion. These encompass the Aachen Aphasia Test (AAT) [9, 16], the ‘Batteria per l’analisi dei deficit afasici’ (BADA) [17], and the ‘Esame Neuropsicologico per l’Afasia’ (ENPA) [18]. However, these tasks include a very small number of stimuli. Normative data are available for the ENPA while the validation of the AAT and the BADA is based on clinical aphasic populations and small samples of healthy Italian participants.

Therefore, the aim of the present study is to develop a new, short, writing to dictation test and to provide robust normative data for the Italian population. Indeed, the test developed by Luzzatti et al. [9] includes a high number of stimuli, some of which (loanwords) were not used for clinical assessment of AD; in addition, normative data were acquired only on 110 healthy participants, coming from the same part of Italy. We also aimed to verify the feasibility of its clinical application on patients suffering from an amnestic Mild Cognitive Impairment (aMCI) and on patients suffering from AD.

## Materials and methods

### Participants

Four hundred and sixteen healthy participants were enrolled in the study. Participants were voluntarily recruited through acquaintances of the authors. All the participants were community dwelling individuals living independently in several districts of Trentino, Veneto, Lombardia, and Umbria (North and Central Italy). Selection criteria included: (i) the absence of clinical and/or anamnestic evidence of current or previous brain pathologies, history of developmental disorders, head trauma, alcoholism or psychiatric disorders requiring pharmacological treatment; (ii) an adjusted score below the national cut-off score at the Montreal Cognitive Assessment (MoCA) [19]. We further enrolled a sample of 28 patients at the Neurocognitive Rehabilitation Center of the Interdepartmental Mind/Brain Center (University of Trento). The patients were divided into two groups according to disease severity: (i) 14 patients with a diagnosis of MCI (8 female; mean age: 72.57 ± 5.17, range: 60–79; mean educational level: 12.78 ± 3.19, range: 8–18; mean MoCA’s raw score = 21.21 ± 3.80, range: 15–30 ); (ii) 14 patients with a diagnosis of probable AD according to NINCDS-ADRDA’s criteria [20] (8 female; mean age: 70.64 ± 7.23, range: 54–82; mean educational level: 10.71 ± 3.97, range: 8–18; mean MoCA’s raw score = 15.78 ± 3.72, range: 11–22). For all patients, a clinical and neuropsychological baseline assessment was performed by an experienced neurologist (CP), and neuropsychologists (ST and BF). Demographic, neuropsychological, and clinical features of the patients are available in the Supplementary Materials. All participants provided informed consent to the study, which was conducted following the ethical guidelines of the local ethics committee (prot. 2024-053) and the Declaration of Helsinki.

### The test

Writing to dictation was tested through three categories of stimuli (see Supplementary Materials):

i) *Words with regular one-sound-to-one letter correspondence*. In this section, the spelling of 40 words with regular shallow orthography was tested.
ii) *Words with unpredictable orthography*. This set of stimuli includes 30 words with an orthography not fully predictable by the application of regular sub-word-level spelling rules [e.g., 10, for the principal features of the Italian orthography].
iii) *Non-words with regular one-sound-to-one letter correspondence*. The last set of items includes 20 regular non-words with a phonological and graphemic structure parallel to that of the regular words. Non-words were matched for length and phonological/orthographic structure with the regular words and were obtained by changing one or two letters (consonant, vowel or both) from a word.

The lexical stimuli (categories i) and ii)) were matched for word frequency and length. The mean word frequency for each section is shown in the Supplementary Materials.

Words and non-words were tested separately. Words were randomised for regularity, word frequency and length; non-words for complexity and length. The examiner read each item aloud, in a neutral voice, and participants had to write in capital letters the heard stimuli. We asked to write in capital letters to avoid confusion, for example between “a” and “o” as final letters. Each item could be repeated once on request, but no feedback was provided. Spontaneous repairs were accepted. For each item, one point was assigned if correctly spelled. Three different scores were computed for regular words (range: 0–40), words with unpredictable orthography (range: 0–30), and non-words (range: 0–20).

### Statistical analyses

Preliminary normality checks on raw scores were performed by both evaluating data distribution (Shapiro-Wilk test), and visually inspecting plots for outliers detection. Eleven participants were excluded from further analysis since their score on the non-word subtest fell at least three standard deviations below the average of the sample. These participants had a mean age of 84.63 ± 3.23 years (range: 81–90), and we hypothesize that possible hearing impairments might have accounted for their performance. After outliers’ removal, an additional analysis did not reveal significant differences between participants with *≤* 5 and 6–8 years of education when spelling non-words (*p* = .458). However, a significant difference between the two groups was found when spelling regular (*p* < .001) and unpredictable words (*p* = .01). Therefore, to improve the statistical accuracy of the test and to avoid unstable estimates, we removed the subgroup of participants with *≤* 5 of education (*N* = 13) from further analysis.

Descriptive statistics were computed based on the performance of three hundred and ninety-two healthy participants with at least 6 years of formal education. Due to non-normal distribution (age: Shapiro-Wilk W = 0.958, *p* < .001; education: Shapiro-Wilk W = 0.943, *p* < .001; MoCA: Shapiro-Wilk W = 0.949, *p* < .001; regular words: Shapiro-Wilk W = 0.574, *p* < .001; words with unpredictable orthography: Shapiro-Wilk W = 0.773, *p* < .001; non-words: Shapiro-Wilk W = 0.830, *p* < .001; global score: Shapiro-Wilk W = 0.860, *p* < .001), a non-parametric correlation analysis (Spearman’s rank correlation coefficient) was performed to test for between-variable associations (age, educational level, and spelling test scores). Sex differences were tested through the Mann-Whitney test (independent sample). Test-retest reliability was investigated by correlating the performance of 20 participants (11 female; mean age: 44.84 ± 18.66, mean education: 15.53 ± 3.73) on the test session and on a retest session three months later. Learning effects between the test and the retest session were assessed via three paired-samples t-tests with Bonferroni’s correction. Regression-based norms were derived using Arcara’s procedure [21], which proved to outperform the traditional methods proposed by Capitani and Laiacona [22]. This method is based on a model selection metric (i.e., Akaike Information Criterion - AIC), which considers all predictors together to avoid the pitfalls of simple regression selection preceding multiple regression analysis. When necessary, transformed demographic variables (logarithmic, inverse or cubic), were employed to identify the strongest association between predictors and raw scores. This follows the standard practice in regression-based normative studies for neuropsychological testing, where scores often have skewed distributions and non-linear relationships with demographic factors. Adjusted values were then computed by adding or subtracting the contribution of each demographic variable for each participant. Finally, Equivalent scores (ES), were obtained using non-parametric tolerance limits of the adjusted scores [23, 24]. This procedure does not yield a unique normality threshold, but two normality thresholds. The outer tolerance limit (oTL), for the lower 5% of the population, grants a 0.95 safety level when declaring that a patient’s performance is impaired. The inner tolerance limit (iTL), for the upper 95% of the population, grants a 0.95 safety level when declaring that a patient’s performance is ‘*normal*’. For the values in between these two limits, we cannot control for the risk of generalizing from a particular sample to the population. This procedure has largely been used for neuropsychological tests in which part of the healthy participants scored at ceiling, and this especially applies to the tests tapping language abilities [23], and then to our test. The Equivalent Score method standardizes regression-adjusted scores into five categories, ranging from 0 to 4. ESs = 0 and 1 meaning ‘defective’ and ‘borderline’, respectively. ES = 2 meaning ‘low-end normal’; ESs = 3 and 4 meaning ‘normal’. A cut-off is identified through the outer one-sided non-parametric lower tolerance limit (oTL) (the highest ascending-order-ranked adjusted scores yielding a safety level *p* *≥* .95 that no more than 5% of the population performs below it). The method thus inferentially addresses a performance as impaired if falling within the ‘worst’ 5% of the normative sample, by keeping the risk of drawing a wrong inference below 5%. To control for inferential errors, the inner one-sided non-parametric lower TL (iTL) is also computed (the lowest observation yielding a safety level p *≥* .95 that at most 95% of the population performs above it). Adjusted scores lower than the oTL and greater than the median are classified as ES = 0 and 4, respectively [24].

To verify the feasibility of the test in clinical practice, we applied the normative procedure to the performance of 14 MCI patients and 14 AD patients. Furthermore, a qualitative analysis of the types of errors made by both the patients and the healthy participants was performed. Finally, to explore the relationship between the test performance and cognitive functioning, we computed Spearman’s rank partial correlation, controlling for MoCA adjusted score, between the writing test adjusted scores and variables representing cognitive function in different domains (i.e., short-term memory, verbal working memory, executive functions, visuo-constructional abilities and verbal fluency). The p-values resulting from correlation analyses were corrected for multiple comparisons using Bonferroni’s method (*а*_*adjusted*_ = 0.05/*k*, with k equal to the number of correlations performed, i.e., *k* = 9).

## Results

The final sample included 392 healthy participants (207 females; age: 55.6 ± 18.6, range: 19–91, educational level: 13.5 ± 3.89, range: 7–27; MoCA adjusted score: 25.40 ± 2.59, range: 19.47-30). Sample stratification for age, education and sex is reported in Table 1.

**Table 1:** Sample stratification. In each cell, the total number of participants (male/female) is reported; “-” = not available

Education → Age ↓	6–8	9–13	> 13	Total
19–29	-	13 (6/7)	44 (21/23)	57 (27/30)
30–39	-	13 (7/6)	21 (9/12)	34 (16/18)
40–49	3 (2/1)	17 (7/10)	22 (10/12)	42 (19/23)
50–59	16 (10/6)	41 (19/22)	20 (10/10)	77 (39/38)
60–69	21 (8/13)	38 (19/19)	20 (11/9)	79 (38/41)
70–79	19 (8/11)	27 (13/14)	19 (8/11)	65 (29/36)
*≥* 80	12 (5/7)	16 (7/9)	10 (5/5)	38 (17/21)
Total	71 (33/38)	165 (78/87)	156 (74/82)	**392 (185/207)**

Descriptive statistics of the three test scores are reported in Table 2. Scores are higher for younger and more educated participants, and for females. All raw scores are available in the Supplementary Materials.

**Table 2:** The raw scores (mean (standard deviation), median and IQR), in the three subtests are reported, with reference to the whole sample and divided for males and females. The results from the comparisons between the male and female groups are also reported, as a *p*-value from a Mann-Whitney test. ‘*’ = *p* < .05

	Overall	Males	Females	Mann-Whitney’s U *p*-value =
Regular words	M = 39.7 (0.66) Me = 40; IQR:1	M = 39.6 (0.75) Me = 40; IQR: 1	M = 39.7 (0.56) Me = 40; IQR: 0	0.012*
Words with unpredictable orthography	M = 29.0 (1.23) Me = 29; IQR: 1	M = 28.8 (1.35) Me = 29; IQR: 2	M = 29.1 (1.11) Me = 29; IQR: 1	0.132
Non-words	M = 18.6 (1.56) Me = 19; IQR: 2	M = 18.4 (1.68) Me = 19; IQR: 2	M = 18.7 (1.45) Me = 19; IQR: 2	0.116

We found positive correlations between the subtests (Spearman’s rank correlation ranged between 0.162 and 0.330. All *ps < 0.001*). A negative association was found between all test scores and age (Spearman’s rank correlations ranged between − 0.458 and − 0.231. All *ps < 0.001*). Conversely, a positive correlation with years of education (Spearman’s rank correlations ranged between 0.06 and 0.424. All *ps < 0.001*), and with MoCA raw score was found (Spearman’s rank correlation ranged between 0.328 and 0.550. All *ps < 0.001*). (see Supplementary Materials). Mann–Whitney U tests revealed a significant effect of sex on regular words (U = 16990; *p* = .012). No significant differences of sex have been found on words with unpredictable orthography and non-words (*ps > 0.05*) (Table 2).

### Test-rest correlations and learning effects between test and re-test sessions

All test-retest correlations of the writing to dictation test were positive and small/medium. Pearson’s rs ranged between 0.270 and 0.360. Learning effects in participants’ performance between the test and the retest session were addressed *via* three paired-samples t-tests corrected with Bonferroni. Participants performed at ceiling when spelling regular words in both the test and retest conditions. Conversely, their performance significantly improved on the retest for words with unpredictable orthography. No significant difference was found for non-words.

### Adjusted and equivalent scores

Results showed significant effects of age, education, and sex across the scores involving words. Specifically, the cubic age, the inverse of education, and the biological sex were the best predictors of regular words. For words with unpredictable orthography, the best-fitting model included the log-transformed age, the inverse of education, and the biological sex. For non-words, only the cubic age emerged as a significant predictor. Correction grids are available in the Supplementary Materials, as well as a spreadsheet to obtain the adjusted and the equivalent scores by entering the participant’s raw scores, age, education level and biological sex. Table 3 reports the adjustment equations, AIC and R^2^ for the three subtests. ES, oTL, iTL and intervals for the scores are shown in Table 4.

**Table 3:** Adjustment equation to transform raw scores into adjusted scores. AS = adjusted score; RS = raw score. Sex: 0 = Male, 1 = Female. In the “AIC” column, the AIC (Akaike Information Criterion) value of the best predictive model is reported. It is a measure of model performance that takes into account both model fit and model complexity (i.e., the number of parameters in the model). The R^2^ column reports the R^2^ value of the best predictive model

Subtest	Equation	AIC	*R* ^2^
*Regular words*	AS = RS - [− 1.03e-06*(cube(Age) − 227095.005) − 3.63 *(inv(Education) − 0.081) + 0.2 *(identity(Sex) − 0.5)]	748	0.139
*Words with unpredictable orthography*	AS = RS - [0.368*(logm100(Age) − 3.696) – 20.7 *(inv(Education) − 0.081) + 0.27 *(identity(Sex) − 0.5)]	1173	0.251
*Non-words*	AS = RS - [− 4.35e-06*(cube(Age) − 227095.005)]	1357	0.246

**Table 4:** Cut-off values delimiting the Equivalent Scores. The inner tolerance limits are: Regular words = 38.615; Words with unpredictable orthography = 27.785; Non-words = 16.239

Subtest → ES ↓	Regular words	Words with unpredictable orthography	Non-words
0 (oTL)	*≤* 38.047	*≤* 27.087	*≤* 15.59
1	/	27.088–28.292	15.60-16.776
2	/	28.293–29.515	16.777–17.998
3	/	29.516–29.866	17.999–18.906
4	/	> 29.866	> 18.906

### Feasibility and qualitative analysis of the writing profile of the clinical population

The total time to complete the task was around fifteen minutes: none of the patients reported discomfort or tiredness due to the length of the test.

When applying the normative data to the MCI patients’ sample, five of them (35%) showed an impaired or borderline overall performance when writing words with unpredictable orthography, while three out of 14 (21%) showed a borderline performance when writing non-words. None of the patients showed difficulties when writing regular words. Only one patient (7%) showed a borderline or pathological performance in more than one subtest. A fine-grained analysis revealed that four patients showed a deficit when writing words with unpredictable orthography, suggesting an impairment of the lexical pathway (*surface dysgraphia*). Conversely, the remaining MCI patients did not exhibit a writing disorder.

When applying the normative data to the AD sample, 9 out of 14 patients (64%), exhibited an impaired or borderline overall performance when writing words with unpredictable orthography. 8 out of 14 patients (57%) showed an impaired or borderline performance when writing non-words and 7 out of 14 (50%), exhibited an impaired performance with words with regular transcription. Moreover, 8 out of 14 (57%) showed a borderline or pathological performance in more than one subtest. In line with Luzzatti et al. [10], our AD sample showed multiple patterns of writing disorder. Indeed, one patient showed a selective deficit for non-words (*phonological dysgraphia*). One exhibited an impaired performance with both words with unpredictable orthography and non-words (*mixed dysgraphia*). Seven more patients showed an undefined pattern of dysgraphia (one patient showed a selective deficit for regular words, four patients showed a pathological performance for regular words and words with unpredictable transcription, one patient for regular words and non-words, and one showed a pathological performance in all three subtests).

As for the healthy participants, a qualitative analysis of the errors revealed different patterns for words and non-words. With words, both orthographic errors (e.g., ‘*arcere’* for ‘*arciere’*) and phonemic substitutions (e.g., ‘*pacco’* for ‘*tacco’*) were observed, with orthographic errors clearly predominating (74%) over phonemic ones (26%). In the case of non-words, errors were predominantly phonemic substitutions (85%), with an occasional trend toward lexicalization of the target stimulus (e.g., ‘*flienzio’* for ‘*filentio’*).

Finally, as for the peripheral graphomotor aspects, none of the patients exhibited allographic errors. No other signs of early peripheral impairment were observed (i.e., spacing or line maintenance, tracts misplacements).

### Correlations

Spearman’s rank partial correlations, controlling for MoCA adjusted score, between our test adjusted scores and variables representing cognitive function in different domains, did not show significant results (r_s_ ranged from − 0.474 to 0.652. All *ps* > 0.05) (see Supplementary Materials).

## Discussion

The aim of the present study was to develop and validate a novel test for the assessment of writing abilities in the Italian population. To provide reliable normative data and regression-based adjustments, considering the influence of demographic factors on writing to dictation performance, 392 healthy participants from North and Central regions of Italy and with at least six years of formal education were included in the final analysis. Results showed that writing to dictation abilities are significantly affected by sex, age, and education level. Indeed, younger participants with higher levels of education performed better across all the three subtests. Females showed higher scores than males across the three subtests, but the difference between males and females reached significance only for regular words. Furthermore, test–retest correlations confirmed the stability of the measure over time, and the absence of substantial learning effects, demonstrating the reliability and feasibility of the test in both clinical and research settings. A significant improvement was observed only in words with unpredictable spelling, probably because controls checked how to write them when they were unsure of. Overall, the normative results indicate that the test captures the expected demographic variability while maintaining strong psychometric properties, thus providing a concise yet comprehensive tool for assessing both lexical and sub-lexical components. Compared to the other single word spelling tasks, our test has been validated on a large sample of Italian healthy participants from various regions of North and Central Italy. Moreover, different combinations of sex, age and education levels are represented. As mentioned, the test requires around fifteen minutes to be administered but includes enough stimuli to identify the presence of a writing disorder. In addition, cut-off scores for words with regular and irregular spelling and non-words are available, allowing for a qualitative and quantitative assessment of lexical and sub-lexical functioning.

When verifying the feasibility of the test on a sample of MCI and AD patients, none of them reported discomfort due to the length of the test. Moreover, results from the MCI group showed that most of the impaired or borderline performance was related to words with unpredictable orthography, while the performance on regular words and non-words was largely preserved. However, the number of patients exhibiting a pathological score when spelling words with unpredictable orthography appears to be insufficient to speculate that this may be an early marker of MCI. On the other hand, AD patients exhibited more severe and heterogeneous impairments, suggesting a wider deterioration in writing abilities. Indeed, they produced errors not only when writing words with unpredictable orthography but also when spelling non-words and regular words. The qualitative analysis of individual errors corroborated these results, revealing that MCI patients can produce lexical errors, whereas AD patients exhibited a wider range of deficits that included patterns of surface, phonological, and mixed dysgraphia. Notably, consistent with Luzzatti and coworkers [10], significant inter-individual differences have been observed among both MCI and AD patients’ performances. Nonetheless, despite the preliminary nature of these findings and the limited sample size of patients, the lack of significant correlations between writing-to-dictation performance and other cognitive domains may indicate that these writing deficits are not merely secondary to general cognitive deterioration but rather represent a specific linguistic aspect of neurodegeneration [10]. Finally, in line with a very recent study by Bednorz et al. [25] showing that handwriting parameters demonstrated lower sensitivity and specificity in identifying patients with MCI compared to classic neuropsychological tests, no graphomotor abnormalities were qualitatively observed in our samples. Moreover, in the present study writing to dictation was assessed in capital letters to ensure response uniformity: in Italian, most nouns end in **a** or **o**, and lowercase cursive handwriting often creates uncertainty when deciding which letter the patient wrote. Cases of peripheral dysgraphia show dissociations between upper- and lower-case writing as well as between print and cursive handwriting. For instance, Ingles et al. [26] reported the case of a 50-year-old woman showing prominent dissociations in her ability to write in lowercase vs. uppercase and print vs. cursive after a left fronto-parietal stroke. However, the patient also exhibited difficulties when spelling words and non-words across all output modalities, suggesting a phonological dysgraphia in addition to the peripheral deficit.

Our test is, however, not exempt from limitations. Firstly, due to the nature of the test itself, participants should be confirmed to have normal hearing before the test is administered. Secondly, regarding sample stratification, the area of recruitment was restricted to Northern and Central Italy: although some participants were originally from Southern Italy, the sample does not include a sufficient number of individuals to adequately represent it. Furthermore, our test is applicable only to individuals with at least six years of formal education and some co-occurrences of age and education levels happen to be poorly represented (e.g., poorly educated individuals aged < 40 years). This should lead to attention when adjusting the raw scores of individuals with these socio-demographic features.

Finally, as for other neuropsychological tests tapping language abilities, a large part of the healthy participants scored at ceiling when performing our test, thus producing a skewed distribution of data and residuals. To overcome this issue, regression-based norms were derived using a stepwise backward elimination [21], which proved to outperform the traditional methods proposed by Capitani and Laiacona [22]. When necessary, transformed demographic variables (logarithmic, inverse or cubic), were employed to identify the strongest association between predictors and raw scores. Furthermore, Equivalent Scores were obtained using non-parametric tolerance limits of the adjusted scores [23, 24]: this procedure identifies two distinct thresholds (the outer and the inner tolerance limits). Only for values falling between these two limits, the risk associated with generalizing from the sample to the population cannot be adequately controlled.

In conclusion, this study provides normative data for a novel writing to dictation test that is psychometrically sound, demographically sensitive, and clinically usable. Results confirm that writing to dictation performance is influenced by age, education, and sex. The feasibility study of our test in the clinical population demonstrates the presence of writing impairments in AD patients, which seems to be independent of deficits in other cognitive domains. These findings underscore the importance of including standardized writing measures in neuropsychological evaluations of cognitive decline. In the spirit of Open Science, test materials, instructions, and cut-off scores are freely available in the Supplementary Materials. Therefore, interested neuropsychologists can use them in both clinical and research settings. Furthermore, the accessibility of the material may allow authors to replicate results.

## Supplementary Information

Below is the link to the electronic supplementary material.

Supplementary Material 1

Supplementary Material 2

Supplementary Material 3

Supplementary Material 4

Supplementary Material 5
