# Supplementary material for: A novel writing to dictation test to investigate lexical and sublexical processes: Italian normative data and preliminary clinical feasibility in Mild Cognitive Impairment and Alzheimer’s disease
Source: Neurol Sci. 2026 Jul 17;47(8):641. doi: 10.1007/s10072-026-09256-1 (PMC13379433; doi:10.1007/s10072-026-09256-1)
Supplement: Supplementary file 1 — Supplementary Material 1 [file 10072_2026_9256_MOESM1_ESM.docx]

**Supplementary Materials**

| **Stimulus** | **Word frequency (mean ± SD)** |
| --- | --- |
| *Regular words*  *-High frequency items*  *-Low frequency items* | 247,60 ± 373,62  487,05 ± 407,20  8,15 ± 4,60 |
| *Words with unpredictable orthography* | 179,33 ± 235,29 |
| *Total* | 218,34 ± 321,44 |

**Table 1**: Word frequencies of the used items.

|  |  | **Age** | **Education** | **MoCA** | **Global score** | **Regular words** | **Words with unpredictable orthography** | **Non-words** |
| --- | --- | --- | --- | --- | --- | --- | --- | --- |
| **Age** | r_s_ | - |  |  |  |  |  |  |
|  | *p* | - |  |  |  |  |  |  |
| **Education** | r_s_ | -.373 | - |  |  |  |  |  |
|  | *p* | < .001 | - |  |  |  |  |  |
| **MoCA** | r_s_ | -.594 | .492 | - |  |  |  |  |
|  | *p* | < .001 | < .001 | - |  |  |  |  |
| **Global score** | r_s_ | -.517 | .388 | .525 | - |  |  |  |
|  | *p* | < .001 | < .001 | < .001 | - |  |  |  |
| **Regular words** | r_s_  *p* | -.357  < .001 | .209  < .001 | .272  < .001 | .531  < .001 | -  - |  |  |
| **Words with unpredictable orthography** | r_s_  *p* | -.231  < .001 | .424  < .001 | .412  < .001 | .626  < .001 | .162  .001 | -  - |  |
| **Non-words** | r_s_ | -.458 | .206 | .400 | .826 | .330 | .212 | - |
|  | *p* | < .001 | < .001 | < .001 | < .001 | < .001 | < .001 | - |

**Table 2**: correlation matrix between the test raw scores, age, education and MoCA’s raw scores. r_s_ = Spearman’s rank correlation coefficient.

|  |  | **Sex** |
| --- | --- | --- |
| **Global score** | U | 16481 |
|  | *p* | .016 |
| **Regular words** | U  *p* | 16990  .012 |
| **Words with unpredictable orthography** | U  *p* | 17560  .132 |
| **Non-words** | U | 17452 |
|  | *p* | .116 |

**Table 3**: Mann-Whitney’s tests to verify the impact of sex on the test’s raw scores. U = Mann-Whitney’s U.

|  |  | **Regular words** | **Words with unpredictable orthography** | **Non-words** |
| --- | --- | --- | --- | --- |
| **Coloured Progressive Matrices** | r_s_ | .323 | .031 | -.161 |
|  | *p* | .153 | .894 | .485 |
| **Digit Forward** | r_s_ | .102 | .34 | -.101 |
|  | *p* | .621 | .089 | .625 |
| **Digit Backward** | r_s_ | .16 | .357 | .144 |
|  | *p* | .454 | .087 | .503 |
| **Corsi Block** | r_s_ | -.033 | -.138 | -.401 |
|  | *p* | .877 | .512 | .047 |
| **Rey-Osterrieth Complex Figure – Immediate recall** | r_s_ | .245 | .097 | -.049 |
|  | *p* | .328 | .703 | .847 |
| **Rey-Osterrieth Complex Figure – Delayed recall** | r_s_ | .236 | .018 | .069 |
|  | *p* | .346 | .943 | .785 |
| **Semantic Fluency** | r_s_ | .652 | -.398 | .128 |
|  | *p* | .113 | .376 | .785 |
| **Phonemic Fluency** | r_s_ | -.34 | .466 | -.36 |
|  | *p* | .455 | .292 | .428 |
| **Frontal Assessment Battery** | r_s_ | .263 | .237 | .316 |
|  | *p* | .184 | .235 | .108 |

**Table 4**: Partial correlation between the writing to dictation subtests and the other cognitive tests. r_s_ = Spearman’s rank correlation coefficient.


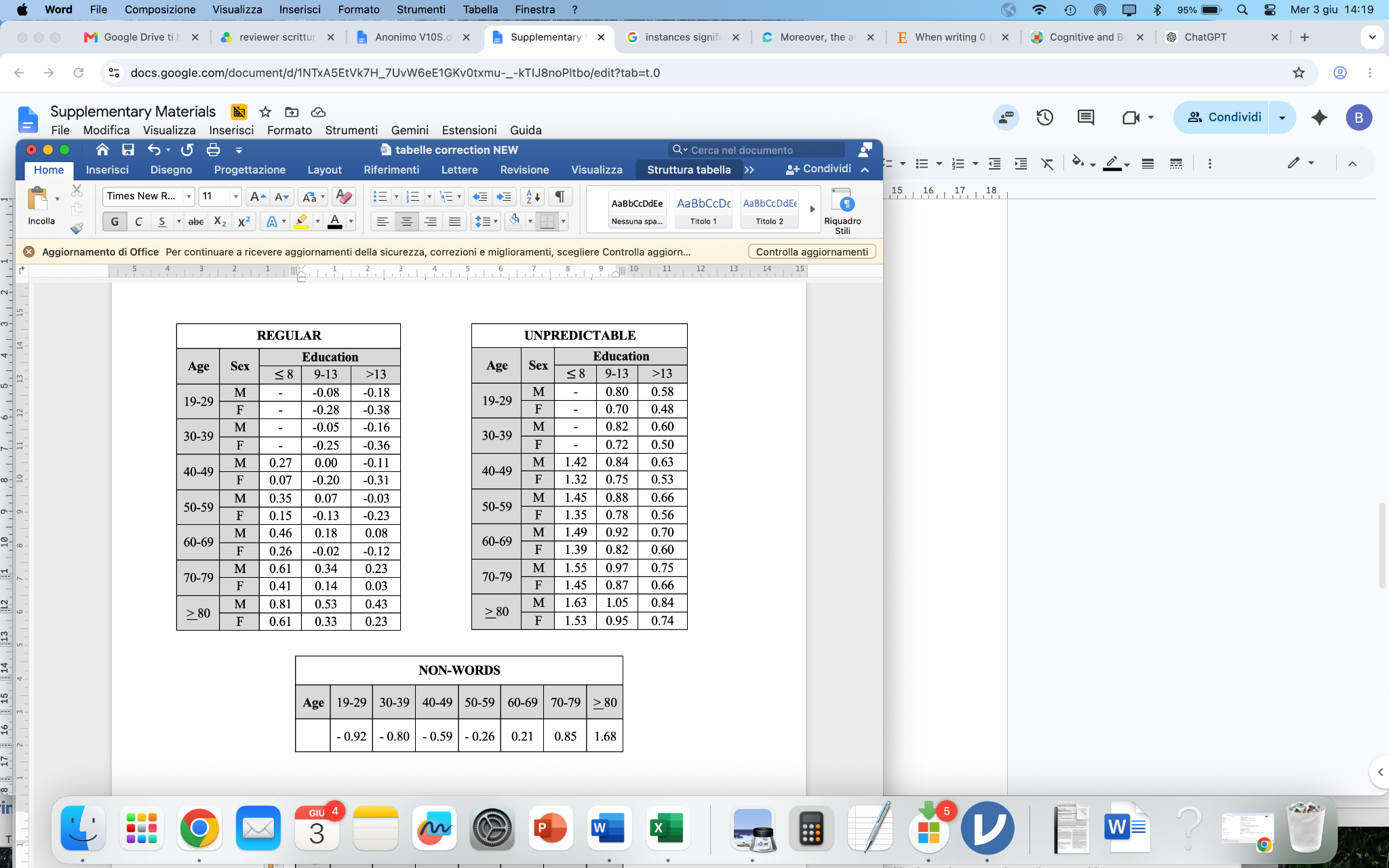


**Table 5**: For each subtest, the correction values are reported based on the combination of age, education, and biological sex. Positive correlation values should be added to the raw score, whereas negative correction values should be subtracted. “-” = not available.
