# Supplementary material for: A novel writing to dictation test to investigate lexical and sublexical processes: Italian normative data and preliminary clinical feasibility in Mild Cognitive Impairment and Alzheimer’s disease
Source: Neurol Sci. 2026 Jul 17;47(8):641. doi: 10.1007/s10072-026-09256-1 (PMC13379433; doi:10.1007/s10072-026-09256-1)
Supplement: Supplementary file 2 — Supplementary Material 2 [file 10072_2026_9256_MOESM2_ESM.pdf]

<u>Protocollo – Parole</u>

L'esaminatore dice "*Ora le detterò alcune parole. Il suo compito è scrivere sul foglio le parole che sentirà. Per favore, scriva in stampatello maiuscolo*"

Su richiesta del soggetto, ogni item può essere ripetuto una seconda volta, ma non oltre.

L'esaminatore deve pronunciare i singoli stimoli in modo chiaro ma neutro, cioè senza accentuare in alcun modo l'eventuale ambiguità ortografica e/o consonanti doppie).

Vengono registrate anche le eventuali correzioni. Non deve essere dato alcun feedback sull'adeguatezza delle risposte.

Punteggio: Si assegna un punto per ogni risposta corretta (in caso di autocorrezioni, viene considerata l'ultima versione).

Item	Stimolo	Trasparenti(T)/ Ambigue(A)	Risposta	Punteggio (0-1)
1	ANGOSCIA	A		
2	MINISTRO	T		
3	CLIMA	T		
4	STRISCIA	A		
5	IGIENE	A		
6	TALLONE	T		
7	RATA	T		
8	FRATELLO	T		
9	CALCAGNO	A		
10	SOLE	T		
11	TELO	T		
12	GENERE	A		
13	COLLA	T		
14	IMBUTO	T		
15	GHIAIA	A		
16	FENOMENO	T		
17	FORMA	T		
18	DEMONIO	A		
19	PIPA	T		
20	SCHIAVO	A		
21	LAVA	T		
22	COLORE	T		
23	GHIACCIO	A		
24	DUNE	T		
25	FABBRO	A		
26	SCOPPIO	A		
27	PRINCIPIO	T		
28	SCHIENA	A		
29	STUFA	T		
30	MODA	T		
31	SPETTRO	A		
32	ARCIERE	A		
33	TACCO	T		
34	CUOIA	A		
35	MACELLO	A		

36	PROBLEMA	T
37	SCOPO	A
38	FUNIVIA	T
39	SACCO	T
40	INQUILINO	A
41	PANNO	T
42	MISANTROPO	T
43	CIELO	A
44	LAMINA	T
45	SILENZIO	T
46	DOMICILIO	A
47	DARDO	T
48	GIUDICE	A
49	OCCASIONE	T
50	CASACCA	T
51	MEDAGLIA	A
52	FARABUTTO	T
53	CIBO	T
54	OLIO	A
55	LANTERNA	T
56	CUORE	A
57	BOCCA	T
58	MAGLIA	A
59	LAMPADARIO	T
60	GARA	T
61	ACQUA	A
62	CORALLO	T
63	OSPEDALE	T
64	SEGNO	A
65	MANO	T
66	VETRO	A
67	PRESIDENTE	T
68	MATRIMONIO	A
69	COLPA	T
70	LIBRO	A

T= /40

A= /30

<u>Protocollo – Non parole</u>

L'esaminatore dice: "*Ora le detterò alcune parole inventate, ossia che non esistono in italiano. Il suo compito è di scrivere su questo foglio quello che, di volta in volta, sentirà dire. Scriva esattamente quello che ha sentito. Posso ripetere una sola volta, se non capisce.*"

In prima battuta invitare il soggetto a <u>scrivere in stampatello</u>.

Su richiesta del soggetto, ogni item può essere ripetuto una seconda volta, ma non oltre.

L'esaminatore deve pronunciare i singoli stimoli in modo chiaro ma neutro, senza accentuare in alcun modo l'eventuale ambiguità ortografica e/o consonanti doppie.

Vengono registrate anche le eventuali correzioni spontanee. Non deve essere dato alcun feedback sull'adeguatezza delle risposte. Eventualmente, dopo che il paziente ha scritto la non parola, chiedere di ripetere e annotare la ripetizione.

Punteggio: Si assegna un punto per ogni risposta corretta (in caso di autocorrezioni, viene considerata l'ultima versione).

Item	Stimolo	Risposta	Punteggio (0-1)
1	LINISTRO		
2	PLIMA		
3	VENòMELO		
4	LORNA		
5	NACCO		
6	PRINCìMIO		
7	NOLMA		
8	FARA		
9	FILèNTIO		
10	CADA		
11	ORPEDALE		
12	LAFA		
13	REFO		
14	GAMPADàLIO		
15	BACCO		
16	NàAMIFA		
17	MUNIVìA		
18	NARPO		
19	LISàNDROPO		
20	LANTèRCA		
			/20
